# What Cachexia-Related Outcomes Are Measured in Lung Cancer Chemotherapy Clinical Trials?

**DOI:** 10.3390/cancers17142309

**Published:** 2025-07-11

**Authors:** Valentina Razmovski-Naumovski, Anthony Tanous, Ross Valaire

**Affiliations:** 1 School of Medicine, Western Sydney University (WSU), Sydney, NSW 2560, Australia; 2 South West Sydney Clinical Campuses, Faculty of Medicine & Health, University of New South Wales (UNSW) Sydney, Sydney, NSW 2170, Australia; 3 Royal Brisbane and Women’s Hospital, Queensland Health, Brisbane, QLD 4006, Australia

**Keywords:** assessment, cachexia, chemotherapy, clinical trials, lung cancer, symptoms, quality of life

## Abstract

Background: Cachexia worsens prognosis, quality of life and chemotherapy treatment compliance of patients with lung cancer. Chemotherapy-induced cachexia has also been implicated in lowered mortality. This study aimed to evaluate the frequency of cachexia-related measures and symptoms as outcomes in lung cancer chemotherapy trial protocols and to examine how key trial characteristics influence them. Method: We conducted a cross-sectional data analysis of randomised controlled chemotherapy trials of lung cancer registered in four public trial registries between 2012 and 2023. Trial outcome measures included overall survival, treatment toxicity/side effects and cachexia-related indicators such as physical activity, weight/body mass index (BMI), dietary limitations, caloric intake and lean muscle mass. Symptom-related outcomes, including appetite loss, diarrhoea, pain, fatigue/insomnia, constipation, nausea, vomiting, dysphagia, dyspnoea and oral mucositis, were also extracted. Additionally, the number and type of performance status and assessment tool were recorded. Data were summarised descriptively. Chi-square tests were used to examine associations between trial outcomes and characteristics including cancer type, trial location, lead investigator/funding source, assessment tools and trial commencement year. A *p* < 0.05 was considered statistically significance. Results: Of the 335 trial protocols (non-small cell (87.2%) and small cell (12.8%)), most were from Europe (50.4%). The trial lead investigator was from industry (56.7%) followed by academia (25.1%). Allied health professional involvement was minimal (0.6%). Trial protocols mostly recorded overall survival (96.4%) and toxicity (83.9%). However, physical activity, weight/BMI, dysphagia, dyspnoea and oral mucositis were recorded in <30%, with dietary limitations, caloric intake and lean muscle mass recorded in <3% of the trials. Measures and symptoms were not associated with cancer type. Trial location was associated with the measures toxicity, physical activity and caloric intake and all symptoms. Lead investigator was associated with the measures toxicity and weight/BMI and all symptoms except for dyspnoea. Performance status and assessment tools were mentioned in 93.4% and 41.8% of the trials, respectively, with significant associations between assessment tools and outcomes, except for weight/BMI, dietary limitations, lean muscle mass, dysphagia and oral mucositis. There was a significant trend with trial commencement year for the measures physical activity (*p* = 0.002) and weight/BMI (*p* = 0.000) and all symptoms, except for appetite loss (*p* = 0.115) and pain (*p* = 0.433). Conclusions: While the reporting of measures and outcomes was generally higher compared to gastrointestinal chemotherapy cancer trials, it still faced significant under-reporting. Assessment tools should include cachexia-specific symptoms to accurately assess the quality of life in patients with lung cancer undergoing chemotherapy clinical trials.

## Simple Summary

Lung cancer is a leading cause of death and is often linked to cachexia, which consists of poor appetite, weight loss and muscle wasting. Chemotherapy can worsen these symptoms, including nausea, fatigue and reduced physical activity. This study looked at how often cachexia-related measures and symptoms were included as outcomes in lung cancer chemotherapy trial protocols and how trial characteristics affected these outcomes. While these outcomes were reported more often than in gastrointestinal cancer trials, they were still under-reported, with related measures appearing in less than 30% of trials. Although 93.4% of trials noted performance status at eligibility, only 41.8% used formal assessment tools. Including cachexia-related outcomes in these tools is crucial to better understand and improve quality of life for patients with lung cancer participating in clinical trials.

## 1. Introduction

Lung cancer is the leading cause of cancer deaths, accounting for 18% of all cancer deaths globally [1]. The disease is associated with a poor prognosis and is often accompanied by the presence of cachexia [2]. Cachexia is a complex “wasting” syndrome prevalent in many advanced diseases, including cancer, chronic obstructive pulmonary disease, renal and heart diseases [3] and, most recently, COVID-19 [4]. Cancer–host interactions manifest cachexia as a combination of various factors including reduced nutrient intake, metabolic changes and inflammatory imbalances, which lead to abnormal glucose, lipid and protein metabolisms [5,6,7]. Cachexia can occur in almost 50% of patients with lung cancer [5], reducing overall survival [8], with approximately 22% of patient deaths from lung cancer being cachexia related [9]. A recent study found that 28% of patients with non-small-cell lung cancer (NSCLC) presented with cachexia at diagnosis, with the prevalence rising to 58% at 12 months [10]. The high incidence of cachexia in lung cancer is thought to be linked to disease progression and the complications from coexisting chronic lung conditions. These factors contribute to persistent systemic inflammation, which can result in appetite and weight loss, malnutrition, sarcopenia and, ultimately, pulmonary cachexia [11].

As chemotherapy is often the first line of treatment for people with lung cancer, there is increasing evidence that chemotherapeutic agents also contribute to the development and progression of cachexia [12]. Chemotherapy-induced toxicities often worsen metabolic and nutritional statuses, due to its non-specific cytotoxicity [13], and worsen symptoms including appetite loss (anorexia), unpleasant changes in the way food tastes, nausea, fatigue and constipation [2,14]. One retrospective study showed that approximately 45% of patients with advanced NSCLC undergoing chemotherapy presented with cachexia at baseline, and this was associated with poor prognosis in patients receiving chemotherapy [2]. Cachexia worsens coexisting health conditions, heightens treatment toxicity and diminishes quality of life, thereby reducing the tolerance and effectiveness of cancer-specific treatments [15]. In patients with small-cell lung cancer (SCLC) and cachexia, it was found that treatment was discontinued early, and prognosis was poor [16]. A study prospectively observed untreated patients > 70 years with advanced NSCLC during initial chemotherapy and reported significant decreases in body mass index (BMI), skeletal muscle index and shuttle walk distance at 12 weeks compared to baseline [17].

Building on the insights from an article highlighting the lack of nutritional patient-reported outcomes (PROs) in clinical trials [18], our recent study found that cachexia-related measures and related symptoms were under-recorded as outcomes in gastrointestinal chemotherapy clinical trials [19]. However, it has not been investigated as to whether this finding extends to other cancers with a poor prognosis, such as lung cancer, given that cachexia impacts this population [20,21]. Thus, the primary aim of this study is to identify what cachexia-related measures and symptoms are recorded as outcomes in lung cancer chemotherapy trials. Our secondary aim is to investigate whether trial characteristics such as lung cancer type, trial location, trial lead investigator/funding source, PRO tools and trial commencement year influence the outcomes recorded and disclose any discernible associations. With overall survival prioritised as a primary endpoint in cancer clinical trials [22], it is hypothesised that cachexia-related measures and symptoms will be under-recorded as outcomes in lung cancer trials. The objective is to raise awareness of the symptom burden experienced by patients undergoing chemotherapy, highlighting the importance of consistently incorporating these factors alongside primary endpoints throughout the trial.

## 2. Methods

We conducted a cross-sectional, meta-research study of randomised controlled trials (RCTs) of chemotherapy in lung cancer between 1 January 2012 to 31 December 2023. It was envisaged that this time period would be adequate to show changes in the clinical trials. Our methods have been previously published [19] and are described as follows:

### 2.1. Search Strategy and Trial Eligibility

This study utilised publicly accessible clinical trial registries of the United States of America [23], Australasia [24], European Union/United Kingdom [25] and China [26]. Other registries were not accessed due to missing trial information and English translation. Search keywords included “chemotherapy” and “lung cancer”. “Cachexia” was not included, as cachexia trials were examined previously [27]. Within the above-mentioned time period, eligibility was based on trial design (Phase II and/or III RCTs; open or blinded), recruitment age (≥18 years) and active/recruiting status (excluding suspended, terminated, withdrawn and prematurely ended trials).

### 2.2. Data Extraction, Items and Synthesis

A standardised data charting form (Microsoft^®^ Excel spreadsheet, v16.8) was used for recording data from each trial. Items for the synthesis included trial location/design/length/status, cancer type [NSCLC and SCLC due to their prevalence [1], year of commencement, lead investigator/funding source (industry, academia, clinic, government), adjuvant therapy, additional medications and allied health professional involvement. Trial outcomes included overall survival, toxicity/side effects, physical activity, weight/BMI, dietary limitations (i.e., restrictions related to certain foods or food sources that were unable to be consumed), caloric intake and lean muscle mass. Symptoms included appetite loss, diarrhoea, pain, fatigue/insomnia, constipation, nausea, vomiting, dysphagia, dyspnoea and oral mucositis. Eligibility (e.g., performance status) and trial assessment tools were extracted, and outcomes within the tools were noted.

### 2.3. Statistical Analysis

Descriptive statistics and frequencies summarised the data for the primary aim. For the secondary aim, data were converted to binary notation for analysis in IBM^®^ SPSS^®^ Statistics (v.29, 2023, Armonk, NY, USA). Pearson’s chi-square/Fisher’s exact tests examined associations between the trial outcomes and the cancer type, trial location, trial lead investigator and PRO tools. The chi-square test of trend assessed associations between the trial length and the number of trials, the outcomes and the trial year. *p* < 0.05 was considered statistically significant.

## 3. Results

### 3.1. Search and Screening Process of the Registered Trials

**Figure 1:**
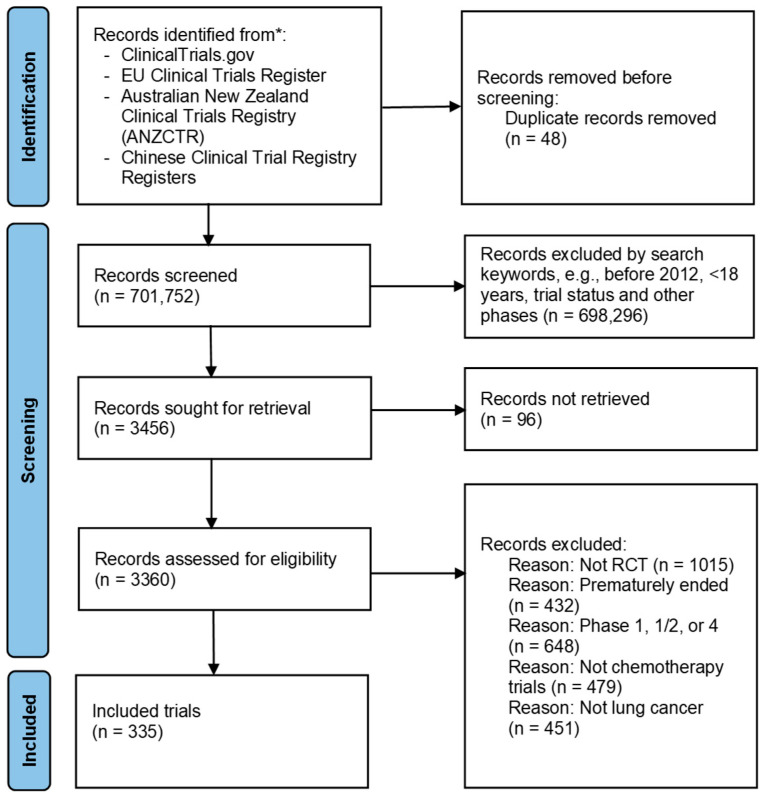
Flow chart for trial selection using the Preferred Reporting Items for Systematic Reviews and Meta-analyses flow diagram (Moher, D., Altman, D.G., Liberati, A. & Tetzlaff, J. PRISMA statement. British Medical Journal 22, 128 (2011)). * https://clinicaltrials.gov/, accessed on 28 June 2025; https://www.clinicaltrialsregister.eu/, accessed on 28 June 2025; https://www.anzctr.org.au/, accessed on 28 June 2025; https://www.chictr.org.cn/indexEN.html, accessed on 28 June 2025.

From 3360 records, 3025 were excluded due to not being RCTs, prematurely ended, Phase I, I/II or IV trials, not chemotherapy trials, not lung cancer trials and commencing before January 2012, resulting in 335 trials included in the final analysis (Figure 1).

### 3.2. Trial Characteristics

There were more NSCLC (87.2%, n = 292) than SCLC (12.8%, n = 43) trials (Table 1). Europe had the most trials (50.4%, n = 169), followed by Asia (28.4%, n = 95), North America (20.6%, n = 69), and Australasia (0.6%, n = 2). Most trials were open/unblinded (70.4%, n = 236), followed by blinded (29.6%, n = 99). The percentage of trials ranged from 4.8% (2013) to 15.5% (2020). The line of chemotherapy was unable to be determined. The lead investigator/funding was dominated by industry (56.7%, n = 190), followed by academia (25.1%, n = 84), clinic (16.7%, n = 56) and government (1.5%, n = 5). Allowed medications were stated in 3% (n = 10) of the trials. Allied health professional involvement was noted in 0.6% (n = 2) of trials. A performance status at eligibility was included in 93.4% (n = 313) of the trials, with the Eastern Cooperative Oncology Group (ECOG) Performance Status Scale the most frequently indicated (82.1%, n = 275; Table 2). Comparatively, specific assessment tools were indicated in 41.8% of trials (n = 140), with the European Organisation for Research and Treatment of Cancer Quality of Life Questionnaire (EORTC QLQ-C30) (30.1%, n = 101) as the most often indicated. Only one assessment tool was indicated in 21.8% (n = 73) of the trials, and two or more were indicated in 20% (n = 67) of the trials.

### 3.3. Trial Outcomes (Measures and Symptoms Reported)

Overall survival and toxicity/side effects were measured most often (96.4%, n = 323 and 83.9%, n = 281, respectively), followed by physical activity (29.3%, n = 98) and weight/BMI (25.4%, n = 85). The least measured were caloric intake (2.7%, n = 9), dietary limitations (1.8%, n = 6) and lean body mass (0.9%, n = 3). For the symptoms, nausea was measured the most (77.0%, n = 258), followed comparatively by vomiting (76.1%, n = 255), diarrhoea (76.1%, n = 255), constipation (75.5%, n = 253), fatigue/insomnia and pain (74.6%, n = 250 and 74.3%, n = 249, respectively). The least measured were appetite loss (44.8%, n = 150), oral mucositis (23.3%, n = 78), dyspnoea (20.0%, n = 67) and dysphagia (17.6%, n = 59) (Table 3).

#### 3.3.1. Associations Between Trial Outcomes and Trial Characteristics

##### Trial Outcomes Across Lung Cancer Type

There were no significant associations of outcome measures with lung cancer type. NSCLC trials reported overall survival (96.9%, n = 283), toxicity (83.9%, n = 285), physical activity (30.5%, n = 89), dietary limitations (2.1%, n = 6), caloric intake (3.1%, n = 9) and lean muscle mass (1.0%, n = 3), as well as the symptoms appetite loss (45.5%, n = 133), diarrhoea (77.4%, n = 226), pain (74.7%, n = 218), fatigue/insomnia (75.7%, n = 221), constipation (77.1%, n = 225), nausea (78.4%, n = 229), vomiting (77.7%, n = 227), dysphagia (18.2%, n = 53), dyspnoea (21.6%, n = 63) and oral mucositis (23.6%, n = 69), more frequently than SCLC trials (Table 3).

##### Trial Outcomes Across Trial Location

Except for overall survival, weight/BMI, dietary limitations and lean muscle mass, the outcome measures and symptoms reported were significantly associated with trial location. As a percentage, trials from Australasia measured overall survival, toxicity/side effects, diarrhoea, pain, fatigue/insomnia, constipation, nausea, vomiting, dyspnoea (all 100%; n = 2) and caloric intake (50%, n = 2) the most. European trials measured physical activity (39.6%, n = 67), dietary limitations (2.4%, n = 4) and appetite loss (53.8%, n = 91) the most. Trials from North America measured dysphagia (23.2%, n = 16) and oral mucositis (31.9%, n = 22) the most and did not measure dietary limitations and lean muscle mass. Trials from Asia reported a lower percentage of outcomes for toxicity/side effects (73.7%, n = 70), caloric intake (2.1%; n = 2), diarrhoea, constipation (both 61.1%, n = 58), pain, fatigue/insomnia (both 60%, n = 57), nausea (63.2%, n = 60), vomiting (61.1%, n = 58) and dyspnoea (13.7%, n = 13) compared to other regions (Table 3).

##### Trial Outcomes Across Lead Investigator

The outcome measures toxicity/side effects (*p* = 0.004), weight/BMI (*p* = 0.000), and symptoms appetite loss (*p* = 0.002), diarrhoea (*p* = 0.005), pain (*p* = 0.008), fatigue/insomnia (*p* = 0.002), constipation (*p* = 0.002), nausea (*p* = 0.006), vomiting (*p* = 0.001), dysphagia (*p* = 0.048) and oral mucositis (*p* = 0.011) were significantly associated with the lead investigator. Industry measured eleven outcomes (physical activity, appetite loss, diarrhoea, pain, fatigue/insomnia, constipation, nausea, vomiting, dysphagia, dyspnoea and oral mucositis) the most compared to three for government (overall survival, toxicity/side effects and weight/BMI) (Table 3).

Significant associations were found between the trial outcomes and PRO tools except with weight/BMI, dietary limitations, lean body mass, dysphagia and oral mucositis, which had *p* > 0.05 (Table 3).

#### 3.3.2. Changes in Outcomes Reported in Trials by Trial Commencement Year

The proportion of cachexia-related outcomes reported in all trials by year (Table 4) showed a significant linear trend for the outcomes physical activity, weight/BMI, diarrhoea, fatigue/insomnia, constipation, nausea, vomiting, oral mucositis, dysphagia and dyspnoea (all *p* < 0.05) in this study. Compared to previous years, outcome measures for the year 2023 are generally less for appetite loss, pain, fatigue, constipation, nausea, vomiting and dyspnoea.

## 4. Discussion

Cachexia is an extremely debilitating condition that impairs the patient’s physical functioning and quality of life and reduces their chances of survival [9]. Much research has focused on cancer cachexia as a syndrome driven primarily by the tumour, often overlooking chemotherapy as a contributing factor [28]. However, many patients with lung cancer begin treatment with chemotherapy, which can exacerbate metabolic imbalances and contribute to the worsening of cachexia through side effects such as oral mucosal damage, diarrhoea, nausea and vomiting [11]. This led us to explore how cachexia-related measures and symptoms were recorded in lung cancer clinical trials, revealing patterns similar to those observed in our previous study on gastrointestinal chemotherapy trials [19].

As expected, overall survival was the most recorded outcome across the clinical trials as it is considered the “gold standard” endpoint in assessing the effectiveness of cancer treatments [22]. However, weight loss/BMI has been found to be significantly associated with overall survival in lung cancer [15,29], with ≥2% weight loss associated with poor overall and progression-free survival [30]. Although higher than our previous study (11.5%) [19], the current study found that approximately 75% of the trials failed to identify body weight and/or BMI as an outcome of the trial. This could be because the National Cancer Institute’s Common Terminology Criteria for Adverse Events (NCI CTCAE) indicates nutritional support for ≥10% weight loss from baseline [31]. Given the ease of frequent measurements and sustained prognostic association, weight measurement should be integrated for future clinical trials of patients with advanced lung cancer [29,30]. The distinction between intentional and unintentional weight loss would further add valuable insight into patient outcomes and help tailor more effective treatment and support strategies.

Due to the multifactorial nature of cachexia, relying on weight loss alone is not sufficient to accurately identify cachexia [32]. Malnutrition, as well as cachexia, have been shown to negatively influence oncological outcomes in patients with NSCLC and should be evaluated before undergoing chemotherapy [33]. Nutritional impact symptoms management is of clinical significance as increased nutritional risk/malnutrition has been reported in 21–45% of patients with lung cancer [13]. In one study, 42% of patients with lung cancer experienced different types of nutritional impact symptoms: appetite loss, vomiting and dysphagia were noteworthy prognostic indicators of malnutrition, cancer cachexia and shorter overall survival and closely related to quality of life [34]. Although higher than our previous study (26.1%) [19], appetite loss was measured in less than half of the trials in this study, with dietary limitations and caloric intake poorly measured (<3%) in the trials. One study found that deteriorating nutritional status in patients with advanced lung cancer was largely due to chemotherapy-induced side effects [35]. In patients with Stage 4 NSCLC, chemotherapy-related anorexia has been linked to significant weight loss and decreased treatment efficacy. These findings underscore the importance of proactive supportive therapy to manage chemotherapy-induced anorexia, prevent malnutrition and weight loss and preserve treatment efficacy [36]. For patients who have cachexia, nutritional interventions, in conjunction with exercise and pharmacotherapy, should be administered to address their muscle mass loss and related symptoms including fatigue [28,37]. Lean muscle mass was the least frequently measured outcome (0.9%), despite its association with higher rates of chemotherapy-induced toxicity in NSCLC [38]. In SCLC, reduced muscle mass has been linked to greater treatment intolerance, lower response rate and poorer prognosis [16]. One study reported that approximately 50% of patients with advanced lung cancer undergoing chemotherapy experienced ongoing loss of muscle mass and muscle function. BMI was a protective factor for functional loss [39], which supports the recording of weight in trials.

Unlike our previous study, fatigue/insomnia, nausea, vomiting, pain, diarrhoea and constipation were recorded in around 75% of the trials, compared to <10% in our previous study [19]. Severe dyspnoea, pain and fatigue are considered the most prevalent and co-occurring symptoms in lung cancer [40,41]: moderate-to-severe dyspnoea has been reported by at least 29% of patients and fatigue by over 50% [14,42]. In contrast, the symptoms dysphagia, dyspnoea and oral mucositis were recorded less frequently (17.6–24%) in this study. Oral mucositis is a side effect of chemotherapy that contributes to additional weight loss and reduction in chemotherapeutic drug dosing [43,44]. Oral hygiene monitoring in people undergoing chemotherapy for lung cancer has been shown to reduce the prevalence and intensity of oral mucositis [44].

Ideally, a multidisciplinary team, consisting of dietitians, exercise physiologists and psychologists, should join the trial’s team to focus on the patient’s physical and psychosocial health throughout the trial [45,46]. However, our study uncovered the dearth of allied health professionals. This is coupled by the lack of allowed medication (3.0%) for the trial patients who undoubtedly experience a high symptom burden from cancer and treatment. This reflects the minimal discussion regarding the symptoms and interventions of cachexia amongst patients and health professionals [47]. Since around 50% of the trials lasted 5 years or more, and with most participants experiencing substantial symptoms during chemotherapy [48], the lack of specialised support during this time allows cachexia to worsen, affecting a person’s response to treatment and quality of life.

More trials were recorded for NSCLC than SCLC, which reflects their prevalence rates (85% and 15%, respectively) [49]. However, there were no significant associations between the outcomes and the type of cancer. This could be due to SCLC’s greater aggressiveness compared to NSCLC, which may leave the symptoms and adverse reactions to treatment largely unchanged between the two [50,51].

Half of the trials were from Europe, reflecting the higher burden of lung cancer there (19.5%) [52] compared to Asia (13.8%) [53]. There were significant associations between the trial location and all the symptoms. This may be partially attributed to the effect of the small sample size of trials from Australasia on the analysis. This may also correspond to a previous study that reviewed the Australian New Zealand Clinical Trials Registry and determined that 45% of trials registered from 2005 to 2017 included PROs, whereas the trials in the Clinicaltrials.gov database determined that use of PRO endpoints were included in 27% of the trials between 2004 and 2013 [54].

Despite having a higher global burden, the number of lung cancer trials was proportionally equal to that of gastrointestinal trials. Previous studies have indicated that lung cancer is comparatively overlooked in cancer research despite its significant share of the cancer burden [55,56], possibly due to the stigma associated with it [57]. In our study, industry-led trials were more prevalent than academic ones, with all of the symptoms being recorded more frequently. This may indicate the level of industry funding available. Nearly all outcomes were associated with lead investigator, except for overall survival, physical activity, dietary limitations, caloric intake, lean muscle mass and dyspnoea. This is most likely due to the small numbers for government-funded trials.

Before starting a trial, performance status identifies at-risk individuals so they can be excluded. The prevalence of poor performance status among lung patients is 34% (provider reported) and 48% (patient reported) [58]. Nearly all studies used the ECOG tool. However, it cannot differentiate patients’ health status prior to developing lung cancer, and subjective factors come into play when assigning a performance scale value to each patient and interpreting trial outcomes [59]. One study found that the poor performance status (measured by ECOG) in patients with SCLC was primarily due to severe conditions directly related to SCLC, like cachexia and vertebral metastasis, as well as other serious comorbidities such as dermatomyositis/polymyositis and pulmonary fibrosis [60]. Therefore, any interpretation of data in these patients must account for this subjectivity, as a wide variation exists in the criteria used to label patients. A cachexia index (CXI) could be used to estimate the risk of cancer cachexia in advanced NSCLC, helping to determine eligibility for studies [61].

Lung cancer patients undergoing chemotherapy face numerous limitations due to symptoms and disruptions to their quality of life, resulting from both the disease and its treatment [51]. In advanced NSCLC, a study showed that cachexia was associated with poorer quality of life (using several assessment tools) and recommended the predictive validity of weight loss [9]. Although most of the assessments in the trials addressed quality of life, many lacked questions related to weight. It is recommended that future assessments in trials include this construct.

Our study results revealed significant associations between most trial outcomes and assessment tools, likely because these outcomes were included in the questionnaires. However, around half of the chemotherapy trials did not record any quality-of-life tools. This indicates that lung cancer chemotherapy trials are not effectively utilising quality-of-life tools to address cachexia-specific symptoms. In a previous study, quality of life was identified as an endpoint in 49% of trials, with the EORTC QLQ-C30 used as an endpoint in 48% of the NSCLC first-line chemotherapy trials. This demonstrated poor quality and variability in the measurement, analysis and reporting of quality of life [62], which is consistent with our findings. A different study reported that 42% of Phase III trials incorporated quality of life as an endpoint for patients with advanced NSCLC undergoing chemotherapy, with the European Organisation for Research and Treatment of Lung Cancer (EORTC QLQ-LC13) being the most used tool (22%), which aligns with our findings. Quality of life was a crucial outcome measure in determining the best standard of care for patients. While chemotherapy demonstrated a positive effect on quality of life and disease-specific symptoms compared to supportive care, it resulted in increased toxicity and a reduction in quality of life in advanced disease. In this patient population, quality of life should be a primary endpoint of treatment to better define meaningful responses in both clinical practice and trials [63]. In another study, the Functional Assessment of Anorexia/Cachexia Therapy (FAACT) and its Anorexia Cachexia Subscale (ACS) performed well compared to other tools, with scores correlated with symptoms and quality-of-life changes typical of cancer cachexia. This supports their validity and value in clinical research for patients with advanced NSCLC, further reinforcing their use as quality-of-life endpoints in clinical trials involving patients with cachexia [64]. PET/CT imaging could also be incorporated into the trial to assess the presence of cachexia and provide additional insights throughout the study. However, its effectiveness in detecting the onset of cachexia in patients with lung cancer is limited [65].

### Strengths and Limitations

A strength of this study is that it reflects the issues discussed in our previous paper [19] regarding cancers that may present with cachexia. We recognise that missing other lung-related cancers and outcomes, such as handgrip strength or cough, may have provided alternative patterns. Although PROs are important for clinical data collection, they are usually analysed after the trial and not shared with the local clinical team. This means they influence future trial designs but are not used by the clinical team to monitor participants during the trial. We stress the need for targeted assessments of cachexia measures and symptoms during trials. This would improve how we measure treatment benefits and side effects, promote better patient–health professional interactions to manage symptoms and support timely and effective chemotherapy. However, variations in diagnostic criteria can significantly influence prevalence estimates of cachexia, potentially undermining the prognostic value of the outcomes examined [66].

The trend for trials in 2023 showed lower outcomes than in previous years, and this may have influenced the analysis. It is anticipated that funding for cancer trials may have been impacted by the COVID-19 pandemic [67]. The line of treatment was not included in this review due to the complexity and variability of chemotherapy regimens across lung cancer subtypes, stages and treatment intents. The lack of English translation and information regarding outcomes in the trials from Asia made it difficult to obtain some data. The number of trials from the Australasian region was much less than the other regions, and thus, these results should be interpreted with caution. The numerous tests to assess the association between the trial outcomes and trial characteristics may have inflated Type-I error rates, and this calls for careful interpretation of these secondary analyses in isolation. Future research could focus on reviewing respective trial publications to evaluate the validity of our findings. Given the specific focus and inclusion criteria of this trial, clinical trials evaluating solely immunotherapies and radiotherapies were not included. However, they could be incorporated into future analyses to assess their impact on outcomes.

## 5. Conclusions

This study highlights the significant unmet challenge of measuring cachexia-related outcomes in lung cancer chemotherapy clinical trials. Future trials should focus on managing the nutritional, physical and psychosocial needs of trial patients, supported by a multidisciplinary cancer team. Comprehensive tools that address all aspects of cachexia are needed to assess quality of life in patients, highlighting the need for further research.

## Figures and Tables

**Table 1:** Characteristics of the included lung cancer clinical trials (n = 335).

Characteristics of Clinical Trials	Number of Trials n (%)
**Cancer type**	
Non-Small Cell	292 (87.2)
Small Cell	43 (12.8)
**Trial location**	
Europe	169 (50.4)
Asia	95 (28.4)
North America	69 (20.6)
Australasia	2 (0.6)
**Trial type**	
Open	236 (70.4)
Blinded	99 (29.6)
**Year commenced (actual or estimated ^a^** **)**	
2012	25 (7.5)
2013	16 (4.8)
2014	18 (5.4)
2015	34 (10.1)
2016	16 (4.8)
2017	31 (9.3)
2018	30 (9.0)
2019	33 (9.9)
2020	52 (15.5)
2021	31 (9.3)
2022	38 (11.3)
2023	11 (3.3)
**Trial length (years)**	
0–1	5 (1.5)
2	32 (9.6)
3	66 (19.7)
4	58 (17.3)
5	66 (19.7)
5–10	99 (29.6)
10+	9 (2.7)
**Lead investigator/funding source**	
Industry	190 (56.7)
Academia	84 (25.1)
Clinic	56 (16.7)
Government	5 (1.5)
**Allowed medication**	
Yes	10 (3.0)
None stated	325 (97.0)
**Allied health professional involvement**	
Yes	2 (0.6)
Not stated	333 (99.4)
**Performance status (at eligibility)**	
Yes	313 (93.4)
Not stated	22 (6.6)
**Number of trials using assessment tools**	
Yes	140 (41.8)
Not stated	195 (58.2)
**Number of trials with**	
1 assessment tool	73
2 or more assessment tools	67

^a^ Start date of trial. Headings are bolded.

**Table 2:** Frequency of performance status and assessment tools in the included lung cancer chemotherapy clinical trials (n = 335).

Performance Status	Frequency (%)	
Eastern Cooperative Oncology Group (ECOG)	275 (82.1)	
WHO Performance Status	27 (8.1)	
Karnofsky Performance Status	6 (1.8)	
Medical evaluation and/or weight loss	5 (1.5)	
None or not stated	22 (6.6)	
**Assessment tool** ^a^	**233**	**Outcomes examined (number) ^b^**
European Organisation for Research and Treatment of Cancer (EORTC QLQ-C30)	101 (30.1)	Physical activity (6), Sleep/fatigue (3), Pain (2), Nausea (1), Vomiting (1), Diarrhoea (1), Constipation (1) and Appetite (1)
European Organisation for Research and Treatment of Lung Cancer (EORTC QLQ-LC13)	61 (18.2)	Pain (3), Dyspnoea (3), Dysphagia (1) and Oral mucositis (1)
EuroQol Group 5 Dimensions 5 Dimension 5 Level (EQ-5D-5L)	23 (6.9)	Physical activity (3), Pain (1)
Functional Assessment of Cancer Therapy–Lung (FACT-L (36 items))	6 (1.8)	Dyspnoea (1), Pain (1), Weight (1), Appetite (1), Sleep (1), Nausea (1), Fatigue (1) and Physical activity (1)
Non-Small Cell Lung Cancer Symptom Assessment Question (NSCLC-SAQ)	4 (1.2)	Pain (2), Fatigue (2), Dyspnoea (1), Appetite (1) and Cough (1)
Lung Cancer Symptom Scale (LCS) ^c^	3 (0.9)	Pain (2), Fatigue (2), Dyspnoea (2), Appetite (2) and Physical activity (1)
European Organisation for Research and Treatment of Cancer Quality of Life Questionnaire in Elderly (EORTC QLQ-ELD14)	3 (0.9)	Physical activity (3), Pain (1)
M. D. Anderson Symptom Inventory Lung Cancer (MDASI-LC)	2 (0.6)	Physical activity (3), Sleep (2), Fatigue (1), Pain (1), Nausea (1), Vomiting (1), Diarrhoea (1), Dry mouth (1), Dyspnoea (1), Constipation (1) and Appetite loss (1)
Others ^d^	14 (4.2)	
Not specified ^e^	16 (4.8)	
Not stated	195 (58.2)	

^a^ Some trials used more than one tool. The listed assessment tools are patient reported. ^b^ Assessment tools and questions were reviewed based on the relevant outcomes studied. ^c^ Consists of observer scale. ^d^ Others used once include EORTC QLQ-CIPN20, EU-SILC, Functional Assessment of Cancer Therapy Trial Outcome Index (FACT-TOI), ICEpop CAPability measure for Adults (ICECAP-A), NCCN Fact Brain Symptoms Index questionnaires, Patient DATA Form, PGIS, PRO-CTCAE, PROMIS, EORTC QLQ LC-29, SF-36, UBQ-C, FAACT and VAS. ^e^ Listed as quality-of-life tool. Headings are bolded.

**Table 3:** Outcomes recorded in the included lung cancer trials (n = 335) and associations between outcomes recorded and cancer type, trial location, trial lead investigator/funding source and assessment tools.

	Variable			Cancer Type			Trial Location					Trial Lead Investigator/Funding Source					Assessment Tools		
Outcome		Yes/No	Number n = 335	Non-Small Cell n = 292	Small Cell n = 43	*p*-Value	Europe n = 169	Asia n = 95	North America n = 69	Australasia n = 2	*p*-Value	Industry n = 190	Academia n = 84	Clinic n = 56	Government n = 5	*p*-Value	Yes n = 140	No n = 195	*p*-Value
**Measures**			n (%)	n (%)	n (%)		n (%)	n (%)	n (%)	n (%)		n (%)	n (%)	n (%)	n (%)		n (%)	n (%)	
Overall survival		Yes	323 (96.4)	283 (96.9)	40 (93.0)	0.200	166 (98.2)	90 (94.7)	65 (94.2)	2 (100)	0.330	183 (96.3)	80 (95.2)	55 (98.2)	5 (100)	0.788	139 (99.3)	184 (94.4)	0.017 *
		No	12 (3.6)	9 (3.1)	3 (7.0)		3 (1.8)	5 (5.3)	4 (5.8)	0 (0.0)		7 (3.7)	4 (4.8)	1 (1.8)	0 (0.0)		1 (0.7)	11 (5.6)	
Toxicity/side effects ^a^		Yes	281 (83.9)	245 (83.9)	36 (83.7)	0.976	148 (87.6)	70 (73.7)	61 (88.4)	2 (100)	0.015 *	170 (89.5)	66 (78.6)	40 (71.4)	5 (100)	0.004 *	131 (93.6)	150 (76.9)	0.000 *
		No	54 (16.1)	47 (16.1)	7 (16.3)		21 (12.4)	25 (26.3)	8 (11.6)	0 (0.0)		20 (10.5)	18 (21.4)	16 (28.6)	0 (0.0)		9 (6.4)	45 (23.1)	
Physical activity		Yes	98 (29.3)	89 (30.5)	9 (20.9)	0.199	67 (39.6)	17 (17.9)	14 (20.3)	0 (0.0)	0.000 *	65 (34.2)	21 (25.0)	11 (19.6)	1 (20.0)	0.127	87 (62.1)	11 (5.6)	0.000*
		No	237 (70.7)	203 (69.5)	34 (79.1)		102 (61.8)	78 (82.1)	55 (87.5)	2 (100.0)		125 (65.8)	63 (75.0)	45 (80.4)	4 (80.0)		53 (37.9)	184 (94.4)	
Weight/BMI		Yes	85 (25.4)	75 (25.7)	10 (23.3)	0.733	49 (29.0)	16 (16.8)	20 (29.0)	0 (0.0)	0.113	66 (34.7)	11 (13.1)	6 (10.7)	2 (40.0)	0.000 *	30 (21.4)	55 (28.2)	0.160
		No	250 (74.6)	217 (74.3)	33 (76.7)		120 (71.0)	79 (83.2)	49 (71.0)	2 (100.0)		124 (65.3)	73 (86.9)	50 (89.3)	3 (60.0)		110 (78.6)	140 (71.8)	
Dietary limitations		Yes	6 (1.8)	6 (2.1)	0 (0.0)	0.343	4 (2.4)	2 (2.1)	0 (0.0)	0 (0.0)	0.644	2 (1.1)	3 (3.6)	1 (1.8)	0 (0.0)	0.533	2 (1.4)	4 (2.1)	0.672
		No	329 (98.2)	286 (97.9)	43 (100.0)		165 (97.6)	93 (97.9)	69 (100)	2 (10.0)		188 (98.9)	81 (96.4)	55 (98.2)	5 (100.0)		138 (98.6)	191 (97.9)	
Caloric intake		Yes	9 (2.7)	9 (3.1)	0 (0.0)	0.243	4 (2.4)	2 (2.1)	2 (2.9)	1 (50.0)	0.001 *	3 (1.6)	4 (4.8)	2 (3.6)	0 (0.0)	0.461	7 (5.0)	2 (1.0)	0.026 *
		No	326 (97.3)	283 (96.9)	43 (100.0)		165 (97.6)	93 (97.9)	67 (97.1)	1 (50.0)		187 (98.4)	80 (95.2)	54 (96.4)	5 (100)		133 (95.0)	193 (99.0)	
Lean muscle mass		Yes	3 (0.9)	3 (1.0)	0 (0.0)	0.504	1 (0.6)	2 (2.1)	0 (0.0)	0 (0.0)	0.497	2 (1.1)	1 (1.2)	0 (0.0)	0 (0.0)	0.876	2 (1.4)	1 (0.5)	0.380
		No	332 (99.1)	289 (99.0)	43 (100.0)		168 (99.4)	93 (97.9)	69 (100)	2 (100.0)		188 (98.9)	83 (98.8)	56 (100)	5 (100.0)		138 (98.6)	194 (99.5)	
**Symptoms**																			
Appetite loss		Yes	150 (44.8)	133 (45.5)	17 (39.5)	0.459	91 (53.8)	33 (34.7)	26 (37.7)	0 (0.0)	0.006 *	102 (53.7)	27 (32.1)	20 (35.7)	1 (20.0)	0.002 *	105 (75.0)	45 (23.1)	0.000 *
		No	185 (55.2)	159 (54.5)	26 (60.5)		78 (46.2)	62 (65.3)	43 (62.3)	2 (100.0)		88 (46.3)	57 (67.9)	36 (64.3)	4 (80.0)		35 (25.0)	150 (76.9)	
Diarrhoea		Yes	255 (76.1)	226 (77.4)	29 (67.4)	0.153	140 (82.8)	58 (61.1)	55 (79.7)	2 (100.0)	0.001 *	157 (82.6)	59 (70.2)	37 (66.1)	2 (40.0)	0.005 *	128 (91.4)	127 (65.1)	0.000 *
		No	80 (23.9)	66 (22.6)	14 (32.6)		29 (17.2)	37 (38.9)	14 (20.3)	0 (0.0)		33 (17.4)	25 (29.8)	19 (33.9)	3 (60.0)		12 (8.6)	68 (34.9)	
Pain		Yes	249 (74.3)	218 (74.7)	31 (72.1)	0.719	137 (81.1)	57 (60.0)	53 (76.8)	2 (100)	0.002 *	154 (81.1)	57 (67.9)	34 (60.7)	4 (80.0)	0.008 *	127 (90.7)	122 (62.6)	0.000 *
		No	86 (25.7)	74 (25.3)	12 (27.9)		32 (18.9)	38 (40.0)	16 (23.2)	0 (0.0)		36 (18.9)	27 (32.1)	22 (39.3)	1 (20.0)		13 (9.3)	73 (37.4)	
Fatigue/insomnia		Yes	250 (74.6)	221 (75.7)	29 (67.4)	0.246	137 (81.1)	57 (60.0)	54 (78.3)	2 (100)	0.001 *	156 (82.1)	57 (67.9)	35 (62.5)	2 (40.0)	0.002 *	128 (91.4)	122 (62.6)	0.000 *
		No	85 (25.4)	71 (24.3)	14 (32.6)		32 (18.9)	38 (40.0)	15 (21.7)	0 (0.0)		34 (17.9)	27 (32.1)	21 (37.5)	3 (60.0)		12 (8.6)	73 (37.4)	
Constipation		Yes	253 (75.5)	225 (77.1)	28 (65.1)	0.089	138 (81.7)	58 (61.1)	55 (79.7)	2 (100.0)	0.001 *	157 (82.6)	58 (69.0)	36 (64.3)	2 (40.0)	0.002 *	126 (90.0)	127 (65.1)	0.000 *
		No	82 (24.5)	67 (22.9)	15 (34.9)		31 (18.3)	37 (38.9)	14 (20.3)	0 (0.0)		33 (17.4)	26 (31.0)	20 (35.7)	3 (60.0)		14 (10.0)	68 (34.9)	
Nausea		Yes	258 (77.0)	229 (78.4)	29 (67.4)	0.110	141 (83.4)	60 (63.2)	55 (79.7)	2 (100)	0.002 *	158 (83.2)	61 (72.6)	37 (66.1)	2 (40.0)	0.006 *	129 (92.1)	129 (66.2)	0.000 *
		No	77 (23.0)	63 (21.6)	14 (32.6)		28 (16.6)	35 (36.8)	14 (20.3)	0 (0.0)		32 (16.8)	23 (27.4)	19 (33.9)	3 (60.0)		11 (7.9)	66 (33.8)	
Vomiting		Yes	255 (76.1)	227 (77.7)	28 (65.1)	0.070	140 (82.8)	58 (61.1)	55 (79.7)	2 (100)	0.001 *	158 (83.2)	60 (71.4)	35 (62.5)	2 (40.0)	0.001 *	128 (91.4)	127 (65.1)	0.000 *
		No	80 (23.9)	65 (22.3)	15 (34.9)		29 (17.2)	37 (38.9)	14 (20.3)	0 (0.0)		32 (16.8)	24 (28.6)	21 (37.5)	3 (60.0)		12 (8.6)	68 (34.9)	
Dysphagia		Yes	59 (17.6)	53 (18.2)	6 (14.0)	0.500	36 (21.3)	7 (7.4)	16 (23.2)	0 (0.0)	0.016 *	43 (22.6)	9 (10.7)	6 (10.7)	1 (20.0)	0.048 *	23 (16.4)	36 (18.5)	0.630
		No	276 (82.4)	239 (81.8)	37 (86.0)		133 (78.7)	88 (92.6)	53 (76.8)	2 (100)		147 (77.4)	75 (89.3)	50 (89.3)	4 (80.0)		117 (83.6)	159 (81.5)	
Dyspnoea		Yes	67 (20.0)	63 (21.6)	4 (9.3)	0.060	42 (24.9)	13 (13.7)	10 (14.5)	2 (100.0)	0.003 *	44 (23.2)	12 (14.3)	10 (17.9)	1 (20.0)	0.383	67 (47.9)	0 (0)	0.000 *
		No	268 (80.0)	229 (78.4)	39 (90.7)		127 (75.1)	82 (86.3)	59 (85.5)	0 (0.0)		146 (76.8)	72 (85.7)	46 (82.1)	4 (80.0)		73 (52.1)	195 (100.0)	
Oral mucositis		Yes	78 (23.3)	69 (23.6)	9 (20.9)	0.696	46 (27.2)	10 (10.5)	22 (31.9)	0 (0.0)	0.004 *	57 (30.0)	12 (14.3)	8 (14.3)	1 (20.0)	0.011 *	28 (20.0)	50 (25.6)	0.228
		No	257 (76.7)	223 (76.4)	34 (79.1)		123 (72.8)	85 (89.5)	47 (68.1)	2 (100.0)		133 (70.0)	72 (85.7)	48 (85.7)	4 (80.0)		112 (80.0)	145 (74.4)	

BMI: body mass index; *p*-value is from a chi-square test or Fisher’s exact test if cell sizes were small. * *p <* 0.05 (bolded) means there is an association between the outcomes and variables. ^a^ Measured by National Cancer Institute Common Terminology Criteria for Adverse Events (NCI CTCAE). Headings are bolded.

**Table 4:** Percentage of outcomes reported in the included lung cancer clinical trials by commencement year.

Year	2012	2013	2014	2015	2016	2017	2018	2019	2020	2021	2022	2023	*p*-Value
**Measures (as %)**													
Overall survival	100	93.7	100	97.1	93.7	93.5	96.7	93.9	98.1	96.8	97.4	90.0	0.925
Toxicity/side effects	84.0	87.5	88.9	85.3	81.3	90.3	83.3	87.9	76.9	77.4	81.6	100	0.795
Physical activity	20.0	12.5	5.6	14.2	25.0	16.1	43.3	33.3	50.0	29.0	36.8	27.3	0.002 *
Weight/BMI	32.0	56.3	61.1	55.9	50.0	45.2	26.7	9.1	7.7	0.0	0.0	9.1	0.000 *
Dietary limitations	4.0	0.0	0.0	0.0	12.5	0.0	6.7	0.0	1.9	0	0.0	0.0	0.060
Caloric intake	4.0	0.0	0.0	0.0	6.3	3.2	6.7	0.0	3.8	3.2	0.0	0.0	0.860
Lean muscle mass	0.0	0.0	0.0	0.0	6.2	0.0	3.3	0.0	1.9	0.0	0.0	0.0	0.536
**Symptoms (as %)**													
Appetite loss	40.0	50.0	50.0	44.1	43.8	58.1	53.3	48.6	53.8	25.8	36.8	9.1	0.115
Diarrhoea	68.0	75.0	77.8	82.4	81.3	77.4	76.7	84.8	78.8	77.4	78.9	90.9	0.001 *
Pain	72.0	62.5	77.8	73.5	81.3	74.2	76.7	78.8	76.9	74.2	78.9	36.4	0.433
Fatigue/insomnia	72.0	68.7	77.8	67.6	81.3	77.4	76.7	87.9	76.9	77.4	78.9	9.1	0.001 *
Constipation	68.0	68.8	77.8	82.4	81.3	74.2	76.7	84.8	78.8	77.4	78.9	9.1	0.001 *
Nausea	72.0	81.3	77.8	82.4	87.5	77.4	76.7	84.8	78.8	77.4	78.9	9.1	0.001 *
Vomiting	72.0	75.0	77.8	82.4	87.5	74.2	76.7	84.8	76.9	77.4	78.9	9.1	0.001 *
Oral mucositis	36.0	43.7	61.1	50.0	43.8	41.9	6.7	15.2	9.6	6.5	0.0	0.0	0.000 *
Dysphagia	28.0	31.3	38.9	47.1	18.8	32.3	16.7	6.1	7.7	0.0	31.6	0.0	0.005 *
Dyspnoea	16.0	0.0	5.6	14.7	6.3	12.9	26.7	30.3	34.6	12.9	0.0	0.0	0.000 *

BMI: body mass index. Headings are bolded; * *p* < 0.05 (bolded) considered significant using chi-square test of trend.

## Data Availability

The original data presented in the study are openly available on ClinicalTrials.gov, EU Clinical Trials Register, Australian New Zealand Clinical Trials Registry and China Clinical Trial Registry at https://clinicaltrials.gov/, accessed on 28 June 2025; https://www.clinicaltrialsregister.eu/, accessed on 28 June 2025; https://www.anzctr.org.au/, accessed on 28 June 2025 and https://www.chictr.org.cn/, accessed on 28 June 2025, respectively.

## Abbreviations

The following abbreviations are used in this manuscript:

CXI	Cachexia index
ECOG	Eastern Cooperative Oncology Group
EORTC QLQ-C30	European Organisation for Research and Treatment of Cancer Quality of Life Questionnaire
FAACT ACS	Functional Assessment of Anorexia/Cachexia Therapy Anorexia Cachexia Subscale
NCI CTCAE	National Cancer Institute’s Common Terminology Criteria for Adverse Events
NSCLC	Non-small-cell lung cancer
PROs	Patient-reported outcomes
RCTs	Randomised controlled trials
SCLC	Small-cell lung cancer
